# MiRNA‐516a promotes bladder cancer metastasis by inhibiting MMP9 protein degradation via the AKT/FOXO3A/SMURF1 axis

**DOI:** 10.1002/ctm2.263

**Published:** 2020-12-21

**Authors:** Yuanyuan Chang, Honglei Jin, Hongyan Li, Jiugao Ma, Zhijian Zheng, Binuo Sun, Yiting Lyu, Mengqi Lin, He Zhao, Liping Shen, Ruirui Zhang, Shuilian Wu, Weiwei Lin, Yongyong Lu, Qipeng Xie, Gang Zhang, Xing Huang, Haishan Huang

**Affiliations:** ^1^ Zhejiang Provincial Key Laboratory of Medical Genetics, Key Laboratory of Laboratory Medicine, Ministry of Education, China, School of Laboratory Medicine and Life Sciences Wenzhou Medical University Wenzhou China; ^2^ The First Affiliated Hospital Wenzhou Medical University Wenzhou China; ^3^ Department of Clinical Laboratory, The Second Affiliated Hospital and Yuying Children's Hospital Wenzhou Medical University Wenzhou China; ^4^ Zhejiang Provincial Key Laboratory of Pancreatic Disease, The First Affiliated Hospital, School of Medicine Zhejiang University Hangzhou China

**Keywords:** bladder cancer, metastasis, migration and invasion, miR‐516a, PHLPP2

## Abstract

**Background:**

Metastasis is the leading cause of death in patients with bladder cancer (BC). However, current available treatments exert little effects on metastatic BC. Moreover, traditional grading and staging have only a limited ability to identify metastatic BC. Accumulating evidence indicates that the aberrant expression of microRNA is intimately associated with tumor progression. So far, many miRNAs have been identified as molecular targets for cancer diagnosis and therapy. This study focused on the role of miR‐516a‐5p (miR‐516a) in BC.

**Methods:**

MiR‐516a expression and its downstream signaling pathway were detected using molecular cell biology and biochemistry approaches and techniques. Fresh clinical BC tissue was used to study the clinicopathological characteristics of patients with different miR‐516a expression. The biological functions of miR‐516a in BC were tested both in vivo and in vitro.

**Results:**

A more invasive BC phenotype was significantly and positively correlated with miR‐516a overexpression in BC patients. MiR‐516a inhibition significantly decreased BC cell invasion and migration in vitro and in vivo. Furthermore, miR‐516a attenuated the expression of PH domain leucine‐rich repeat‐containing protein phosphatase 2 protein and inhibited *SMAD‐specific E3 ubiquitin protein ligase 1* transcription by activating the AKT/Forkhead box O3 signaling pathway, which stabilized MMP9 and slowed down its proteasomal degradation, ultimately promoting BC motility and invasiveness.

**Conclusions:**

Our findings reveal the crucial function of miR‐516a in promoting BC metastasis, and elucidate the molecular mechanism involved, suggesting that miR‐516a may be a promising novel diagnostic and therapeutic target for BC.

## Graphical abstract

1. For the first time, the potential mechanisms underlying the critical role of miR‐516a in bladder cancer metastasis is revealed.
2. MiR‐516a promotes migration and invasion of human bladder cancer cells by targeting PHLPP2 and subsequently inhibiting SMURF1‐mediated MMP9 protein degradation.
3. Based on the metastatic role of miR‐516a in bladder cancer, our findings provide promising targets for bladder cancer therapy.

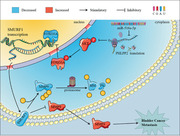

**Abbreviations**
3′‐UTR: 3′‐untranslated region
BAF: bafilomycin A1
BC: bladder cancer
C19MC: chromosome 19 microRNA cluster
CHX: cycloheximide
FBS: fetal bovine serum
FOXO3A: Forkhead box O3
IGF‐1: insulin‐like growth factor‐1
MIBC: muscle‐invasive bladder cancer
NMIBC: Nonmuscle‐invasive bladder cancer
PHLPP2: PH domain leucine‐rich repeat‐containing protein phosphatase 2
SMURF1: SMAD‐specific E3 ubiquitin protein ligase 1

## 1 INTRODUCTION

Reports from the International Agency for Research on Cancer (IRAC) in 2019 and 2020 showed that bladder cancer (BC) ranks fourth in the incidence of male malignancies and is one of the most common malignant tumors of the urinary system.^1^, ^2^ Nonmuscle‐invasive bladder cancer (NMIBC) is present in approximately 75% of patients at the time of diagnosis, and the remaining cases present muscle‐invasive bladder cancer (MIBC) or a metastatic disease.^3^ However, NMIBC patients are at a high risk of progressing to invasive bladder tumor.^4^ Patients with poor prognosis often suffer from the invasive form, and in cases of distant metastasis, the 5‐year survival rate is only 6%.^5^ MIBC is responsible for almost all deaths from BC.^6^ However, current treatments for metastatic BC are ineffective, and the current grades and stages do not accurately predict early metastasis. Therefore, new molecular markers that can predict the recurrence and metastasis of BC are especially important, and novel treatments to combat invasive BC based on identified targets are urgently needed.

MiR‐516a is located on the chr19q13 belonging to the chromosome 19 microRNA cluster (C19MC), the second‐largest imprinted miRNA cluster.^7^, ^8^, ^9^ Poor prognosis in various tumors is often connected with the overexpression of C19MC miRNAs.^10^ One member of this cluster, miR‐519d, is overexpressed in hepatocellular carcinoma cells, promoting cell proliferation and invasion and inhibiting apoptosis.^11^ In addition, miR‐516a‐3p is linked to higher aggressiveness of breast cancer.^12^ Our previous report showed that miR‐516a‐5p is significantly upregulated in human BC tissues and BC cell lines, and that its suppression attenuates the anchorage‐independent growth of BC cells and xenograft tumor growth.^13^ However, the biological role of miR‐516a in the metastasis of BC has not yet been explored.

As a tumor‐suppressor phosphatase, PH domain leucine‐rich repeat‐containing protein phosphatase 2 (PHLPP2) is an important regulator of cellular homeostasis.^14^ Its expression and function are altered in a variety of malignancies.^15^, ^16^ Specifically, PHLPP2 is downregulated in many tumors, and this phenomenon is associated with tumorigenesis.^17^, ^18^, ^19^, ^20^ However, our previous study demonstrated that PHLPP2 is upregulated by p27‐mediated activation of c‐Jun, which influences the regulation of p62 transcription by c‐Jun. This leads to autophagy and autophagy‐dependent degradation of MMP2, ultimately inhibiting human BC cell invasion.^21^ Our more recent study suggested that miR‐516a may influence BC cell metastasis by targeting the 3′‐untranslated region (3′‐UTR) of *PHLPP2*, thereby decreasing the level of PHLPP2 protein. This work revealed a novel mechanism involved in the downregulation of PHLLP2 in BC cells. In addition, our findings further deepened the understanding of the role of PHLPP2 in cancer metastasis.

This study demonstrated that miR‐516a plays an important role in BC metastasis, and the underlying molecular mechanism was partly elucidated. Our results showed that miR‐516a was upregulated in both T24T cell line and invasive BC tissues. Furthermore, we examined the role of miR‐516a in BC cell migration and invasion in vitro and nude mouse lung metastasis in vivo, and showed that PHLPP2 was a direct and functional target of miR‐516a in BC cells. Further experiments showed that miR‐516a inhibited PHLPP2 expression, which in turn activated the AKT/Forkhead box O3 signaling axis to decrease SMAD‐specific E3 ubiquitin protein ligase 1 (SMURF1) expression, eventually slowing down MMP9 protein degradation and promoting BC metastasis.

## 2 RESULTS

### 2.1 MiR‐516a is upregulated in BC and promotes BC metastasis both in vitro and in vivo

**FIGURE 1:**
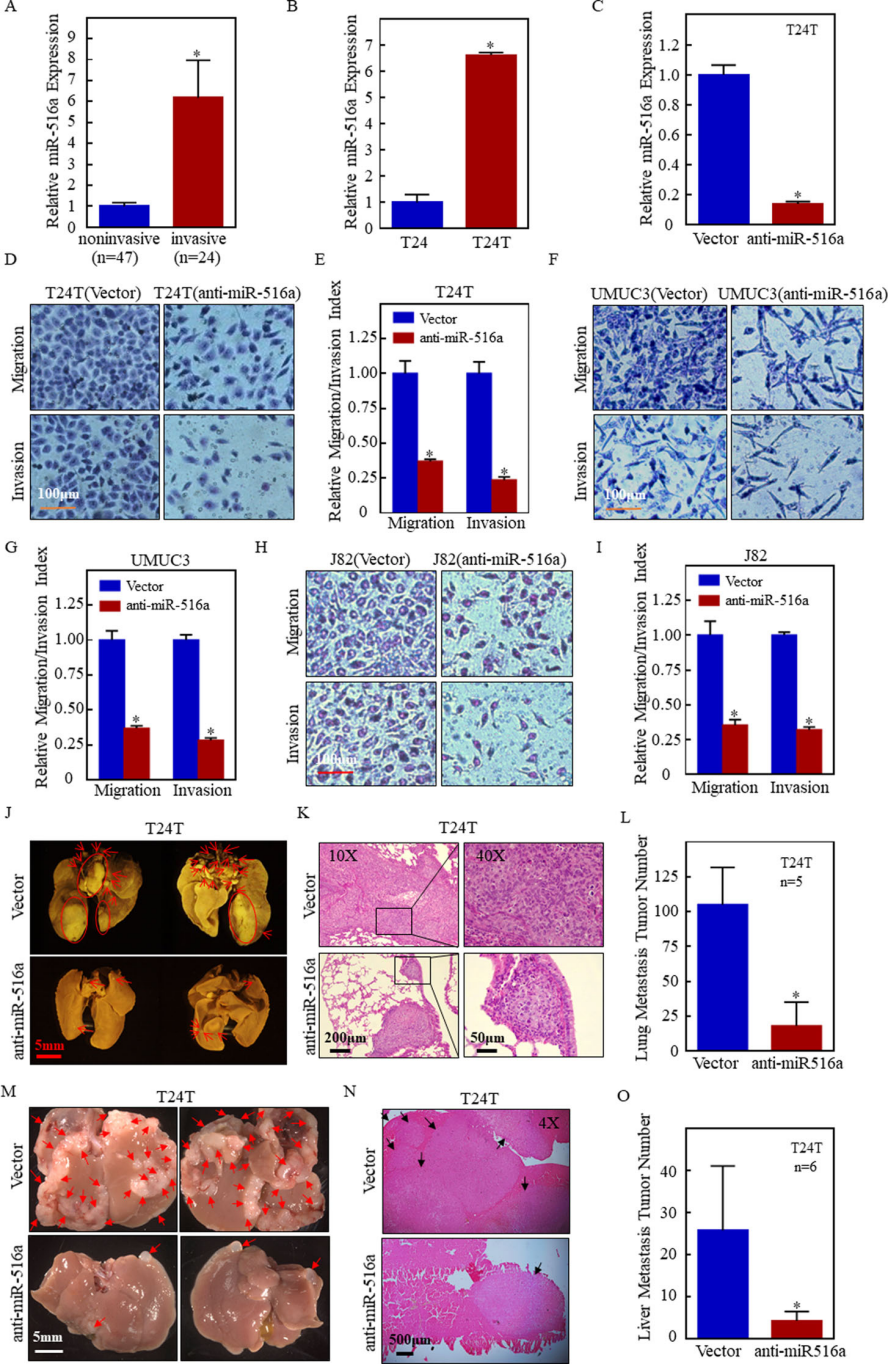
Suppression of miR‐516a inhibited BC cell metastasis in vitro and in vivo. (A) Real‐time PCR was performed to detect miR‐516a expression in human‐invasive BC tissues and noninvasive BC tissues. (B) Real‐time PCR was performed to detect miR‐516a expression in T24 and T24T cell lines. (C) Inhibitory efficiency of miR‐516ain T24T cells was verified by real‐time PCR. (D, F, and H) Transwell assay performed in transfected T24T/UMUC3/J82 cells to evaluate cell migration and invasion ability. Scale bar: 100 μm. (E, G, and I) Graphical representation of panels D, F, and H and statistical analysis. (J‐L) T24T (vector) and T24T (anti‐miR‐516a) cells were injected into nude mice through the tail vein. Images of nude mouse lungs taken using Zeiss SteREO Discovery v.20 (J). (K) Metastatic colonization was confirmed using H&E staining. (L) Lung metastases were counted after post‐fixation with neutral‐buffered formalin/Bouin's solution fixative. The histogram indicates mean ± SD of the number of lung metastases from five mice in each group. (M‐O) T24T (vector) and T24T (anti‐miR‐516a) cells were injected into nude mice through the spleen. Zeiss SteREO Discovery v.20 was used to take the images of nude mice livers (M). (N) Metastatic colonization was confirmed using H&E staining. (O) Liver metastases were counted after post‐fixation with 4% PFA. The histogram indicates mean ± SD of the number of liver metastases from six mice in each group

The leading cause of death from cancer is metastasis,^22^ but the mechanisms that promote the progression from a carcinoma in situ to a metastatic cancer remain elusive. Our ability to improve therapeutic and diagnostic approaches is based on a deeper understanding of the mechanisms regulating tumor metastasis. miR‐516a expression was significantly higher in human‐invasive BC tissues than in noninvasive BC tissues (Figure 1A and Table S1). In addition, the relationship between miR‐516a expression and metastatic status in BC patients was investigated using the TCGA database. The results indicated that miR‐516a expression was increased in patient with lymph node metastasis in comparison to that with no regional lymph node metastasis patient tissues (Figure S1). Therefore, the next step was to evaluate whether miR‐516a overexpression might contribute to a metastatic BC phenotype. The T24/T24T system is a mature model of BC, because T24 cells have limited ability to metastasize, whereas the derivative cell line T24T has a significant metastatic capacity. Accordingly, the T24/T24T system provides an ideal model for studying the molecular mechanisms regulating BC metastasis.^23^, ^24^ First, the expression of miR‐516a in the two cell lines was measured. As shown in Figure 1B, miR‐516a was remarkably higher in T24T than in T24, which was consistent with an association between miR‐516a and BC migration and invasion. Next, a sponge inhibitor targeting miR‐516a (anti‐miR‐516a) was transfected into T24T cells to investigate the mechanism used by miR‐516a to regulate the metastasis of BC cells, and the transfection efficiency was monitored by real‐time PCR (Figure 1C). UMUC3 and J82 cell lines in which miR‐516a was stably inhibited are described in a previous study.^13^ Our functional experiments suggested that the inhibition of miR‐516a decreased the number of BC cell migration and invasion in T24T, UMUC3, and J82 in vitro (Figure 1D‐I).

Next, T24T (transfected with a vector) and T24T (transfected with anti‐miR‐516a) cells were individually injected into nude mice through the tail vein or spleen to evaluate the metastatic ability. In accordance with in vitro study, the inhibition of miR‐516a significantly reduced the degree of lung metastases (Figure 1J‐L and Table S2) as well as liver metastases (Figure 1M‐O and Table S3), compared to the control vector in nude mice. Together, these findings demonstrated that miR‐516a promoted metastasis in human BC cells.

### 2.2 Suppression of PHLPP2 plays a key role in miR‐516a‐mediated promotion of BC cell migration and invasion

**FIGURE 2:**
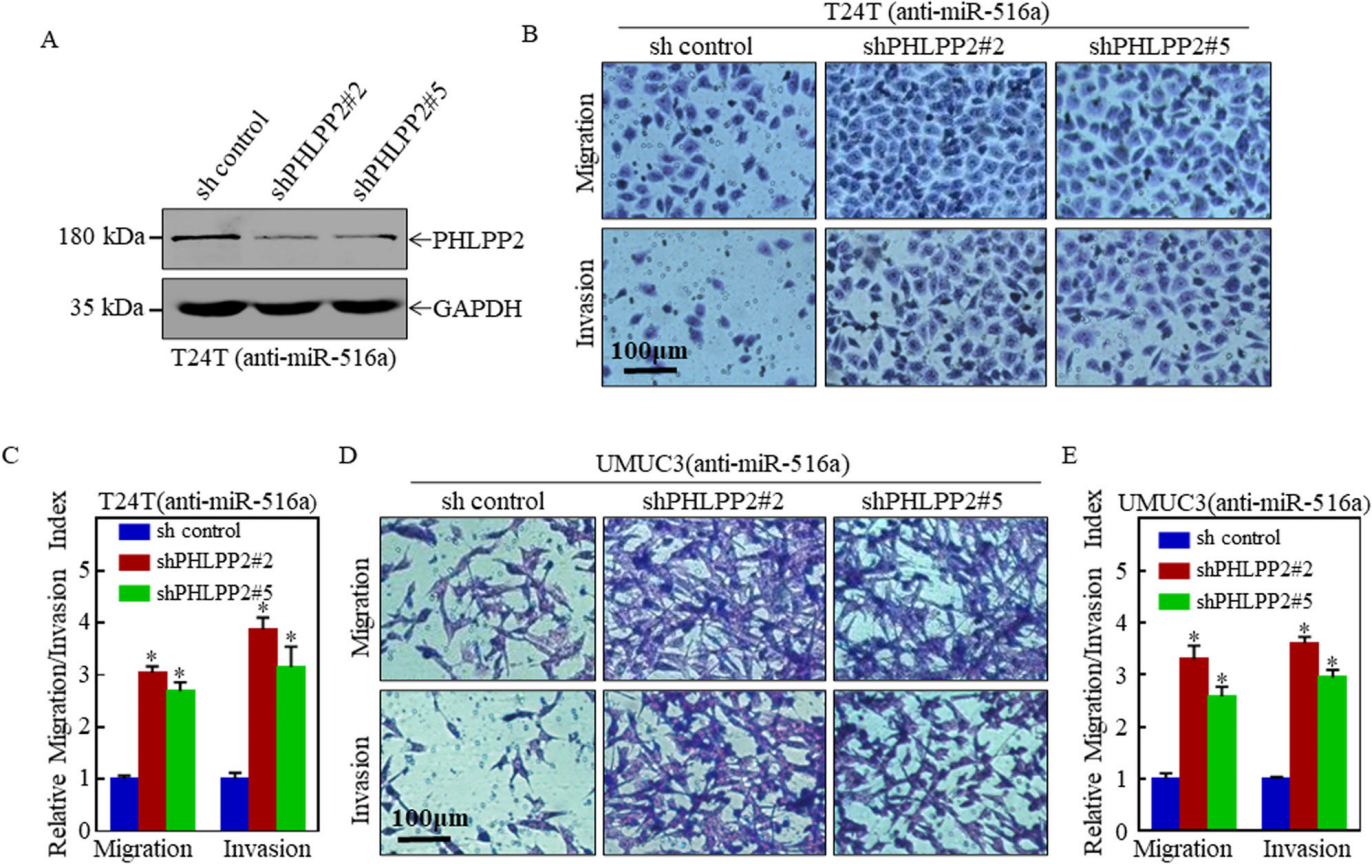
PHLPP2 knockdown plays an essential role in miR‐516a‐induced migration and invasion of BC cells. (A) Knockdown efficiency of PHLPP2 was confirmed in T24T (anti‐miR‐516a) cells by western blot. (B and D) Transwell assay in indicated cells to evaluate the role of PHLPP2 in miR‐516a‐promoted BC cell migration and invasion. Scale bar: 100 μm. (C and E) Graphical representation of panels B and D and statistical analysis

Our previous research revealed that miR‐516a binds the 3′‐UTR of PHLPP2 in UMUC3 and J82 cells.^13^ Thus, we further verified the binding of miR‐516a to the 3′‐UTR of PHLPP2 in T24 and T24T cells (Figure S2). To determine whether PHLPP2 mediates the metastatic role of miR‐516a, PHLPP2‐silenced T24T (anti‐miR‐516a) cells, as well as UMUC3 (anti‐miR‐516a) cells,^13^ were used for further functional analysis. Results showed the silencing of PHLPP2 promoted migration and invasion of both T24T (anti‐miR‐516a) and UMUC3 (anti‐miR‐516a) cells (Figure 2B‐E). Collectively, suppression of PHLPP2 promotes miR‐516a‐mediated BC cell migration and invasion.

### 2.3 MiR‐516a promotes migration and invasion of BC cells through an autophagy‐independent pathway

**FIGURE 3:**
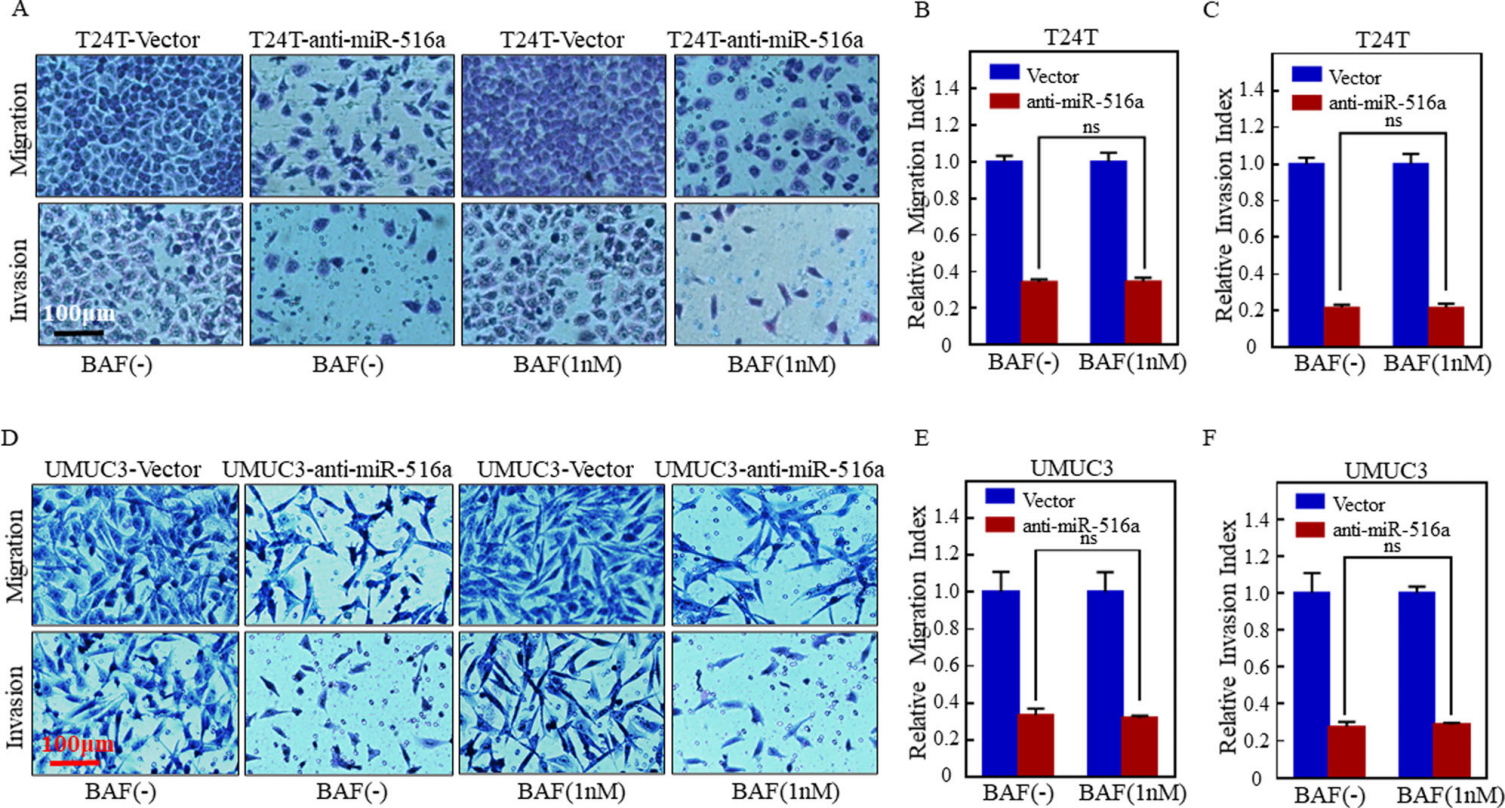
BAF did not affect the promotion of migration and invasion exerted by miR‐516a. (A and D) Transwell assay was performed in transfected T24T/UMUC3 cells treated with or without BAF (1 nM) to evaluate cell migration and invasion. Scale bar: 100 μm. (B, C, E, and F) ns indicates no significant difference between the vehicle‐ and BAF‐treated groups (*P *< .05)

A previous study revealed a connection between autophagy and BC cell growth mediated by miR‐516a.^13^ To determine whether autophagy participates in the regulation of migration and invasion of BC cells, anti‐miR516a‐transfected T24T or UMUC3 cells as well as their control cells were individually treated with an autophagy inhibitor bafilomycin A1 (BAF). Transwell assay revealed that migration and invasion by T24T cells were not affected by BAF treatment (Figure 3A‐C); similar results were also obtained in UMUC3 (Figure 3D‐F). Hence, miR‐516a promotes the migration and invasion of BC cells in an autophagy‐independent manner.

### 2.4 MMP9 is a downstream effector of miR‐516a that promotes migration and invasion of BC cells

**FIGURE 4:**
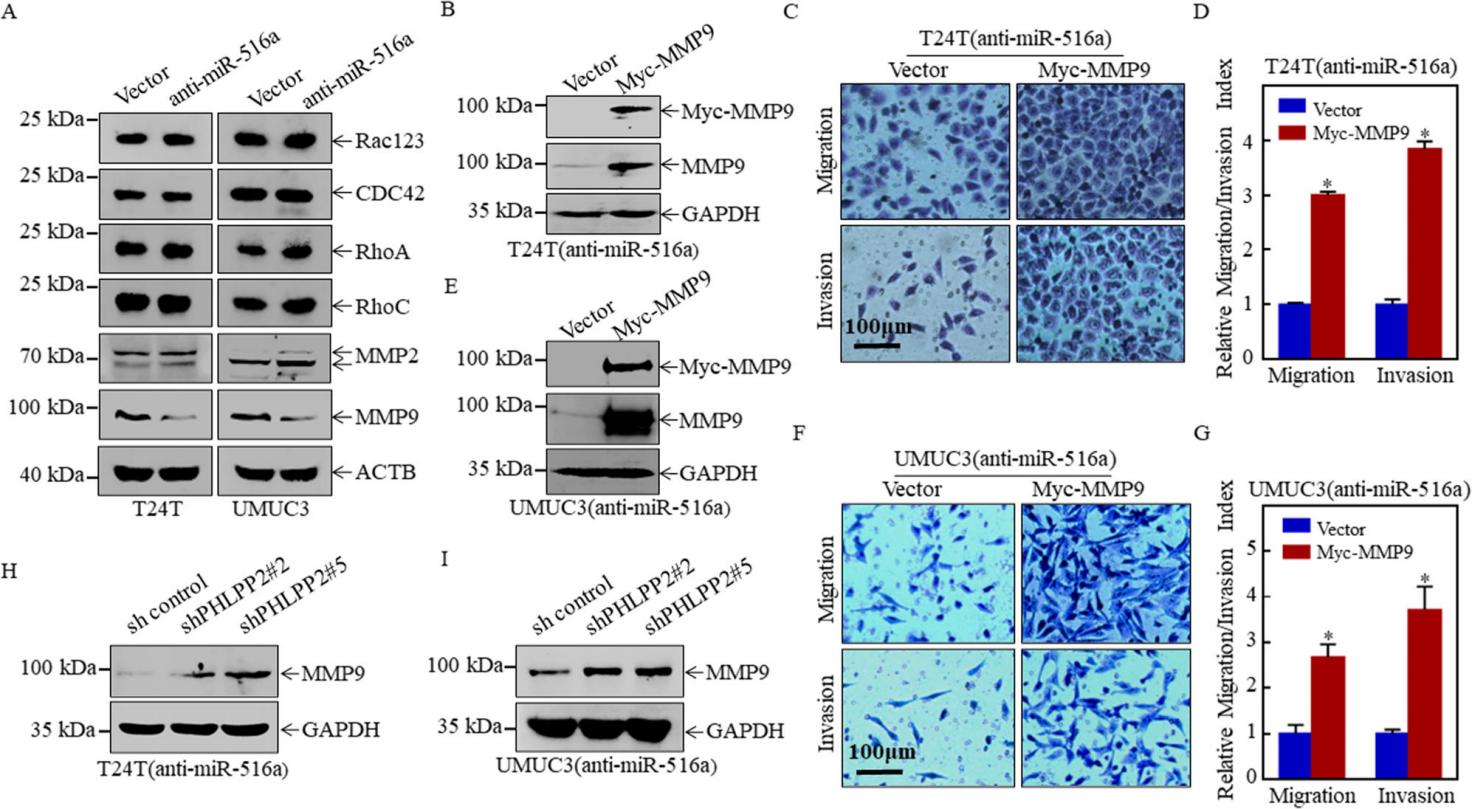
MMP9 played a major role in miR‐516a‐mediated promotion of BC metastasis. (A) Effect of miR‐516a on the protein expression of Rac123, CDC42, RhoA, RhoC, MMP2, and MMP9 in T24T and UMUC3 cells by western blotting. Cells were seeded at the specified densities and starved in 0.1% FBS DMEM or 1:1 (DMEM:F12) for 12 h. Subsequently, the cells were incubated in 10% FBS DMEM or 5% 1:1 (DMEM: F12) for an additional 12 h, and then the cell lysates were subjected to western blotting. (B and E) Overexpression of Myc‐MMP9 in T24T/UMUC3 (anti‐miR‐516a) cells was confirmed by western blot. (C, D, F, and G) Transwell assay was performed in transfected T24T/UMUC3 (anti‐miR‐516a) cells after ectopic expression of Myc‐MMP9. (H and I) The cells indicated were lysed and subjected to western blotting to monitor MMP9 protein expression

Increasing studies showed that both the Rho family and MMP family, including Rac1/2/3, CDC42, RhoA, RhoC, MMP2, and MMP9, are highly expressed in a variety of tumor tissues and are directly correlated with tumor migration and invasion.^25^, ^26^, ^27^, ^28^, ^29^ Thus, we measured the expression of these proteins in miR‐516a‐suppressed T24T and UMUC3 cells as well as the corresponding controls, and found only MMP9 was consistently downregulated in both cell lines after miR‐516a inhibition (Figure 4A). This result implied that the ability of miR‐516a to promote BC cell migration and invasion might be mediated by MMP9.

To characterize the role of MMP9 in miR‐516a‐promoting BC cell migration and invasion, a MMP9 overexpression plasmid or control plasmid was stably transfected into T24T/UMUC3 (anti‐miR‐516a) cells. Stable T24T (anti‐miR‐516a/Myc‐MMP9) and UMUC3 (anti‐miR‐516a/Myc‐MMP9) and their corresponding scrambled‐vector controls were established and identified, as shown in Figures 4B and 4E. Overexpression of MMP9 restored migration and invasion in miR‐516a‐silenced cells, as determined by transwell assay (Figures 4C, 4D, 4F, and 4G). Moreover, MMP9 was upregulated in T24T (anti‐miR‐516a/shPHLPP2 #2 & #5) cells compared to T24T (anti‐miR‐516a/sh control) cells. Similar results were obtained in UMUC3 cells (Figures 4H and 4I). These results demonstrated that the miR‐516a‐PHLPP2 axis facilitated migration and invasion by upregulating MMP9.

### 2.5 MiR‐516a/PHLPP2 cascade regulates MMP9 protein degradation in a proteasome‐dependent manner

**FIGURE 5:**
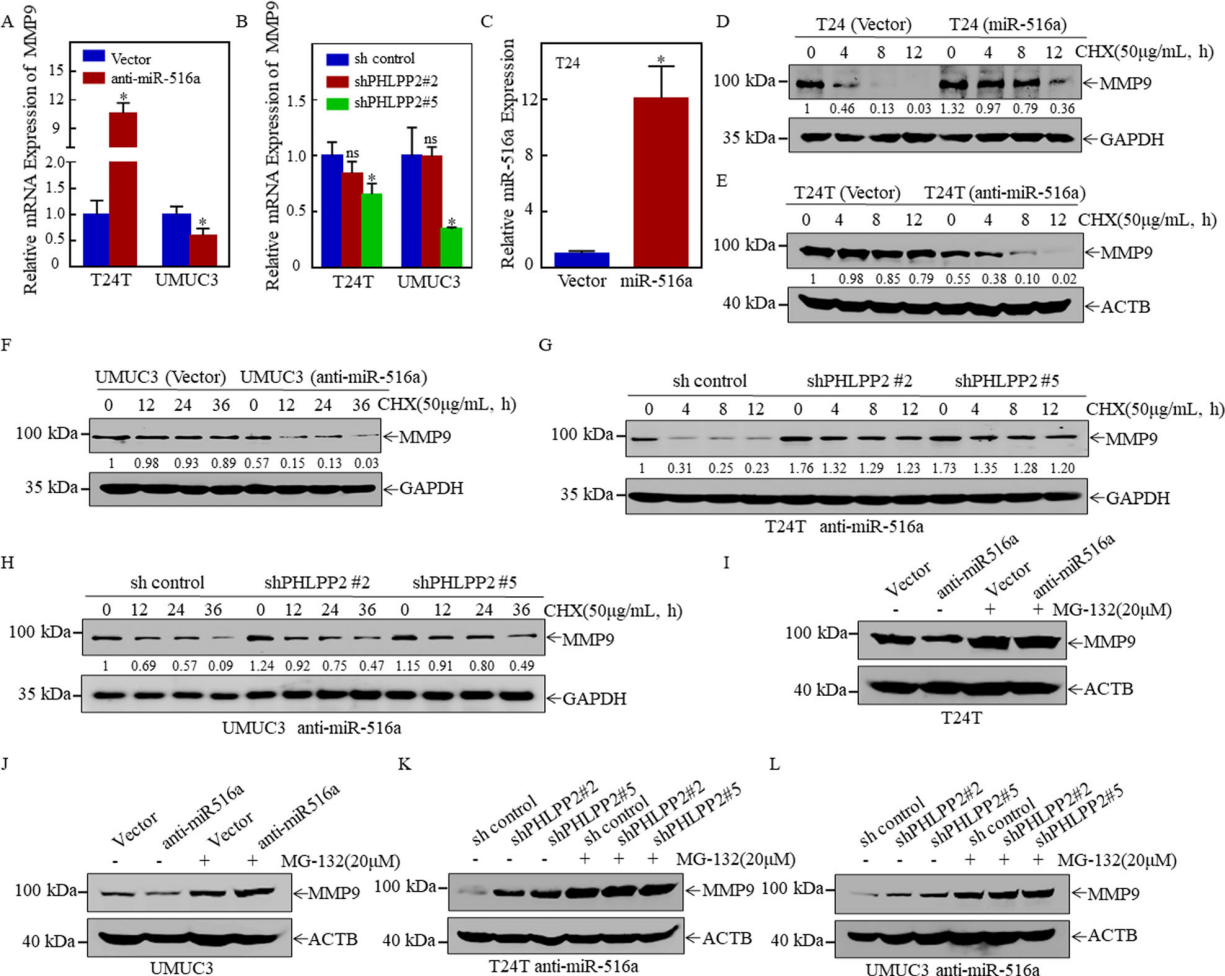
MiR‐516a stabilized MMP9 by inhibiting its degradation by the proteasome. (A) *MMP9* mRNA expression in T24T/UMUC3 (vector) and T24T/UMUC3 (anti‐miR‐516a) cells, as determined by real‐time PCR. (B) *MMP9* mRNA expression in cells indicated, as determined by real‐time PCR. (C) Real‐time PCR was performed to detect miR‐516a expression after ectopic expression of miR‐516a in T24 cells. (D) Rate of MMP9 degradation in T24 (vector) and T24 (miR‐516a) cells, as determined by western blotting. (E and F) T24T/UMUC3 (vector) and T24T/UMUC3 (anti‐miR‐516a) cells were treated with CHX (50 μg/mL) and harvested at the indicated times. The cell lysate was subjected to western blotting with the indicated antibodies. (G and H) Rate of MMP9 degradation in T24T/UMUC3 (anti‐miR‐516a/sh control), T24T/UMUC3 (anti‐miR‐516a/shPHLPP2 #2), and T24T/UMUC3 (anti‐miR‐516a/shPHLPP2 #5) cells, as determined by western blotting. (I‐L) Cells were treated with MG‐132 for 10 h before harvest; protein accumulation was observed by western blotting

Forementioned results suggested MMP9 protein expression was consistently downregulated in T24T and UMUC3 cells after inhibition of miR‐516a expression. However, MMP9 mRNA expression was observed to be upregulated in anti‐miR‐516a T24T cells, whereas it was reduced in anti‐miR‐516a UMUC3 as well as T24T (shPHLPP2 #2 & #5) and UMUC3 cells (Figures 5A and 5B). These results suggested that the miR‐516a/PHLPP2 pathway regulates MMP9 expression at the non‐mRNA level. Next, the effect of miR‐516a on MMP9 protein degradation was assessed. Cycloheximide (CHX) is a protein synthesis inhibitor that interferes with translocation during protein synthesis and hinders translation. The addition of CHX at specific times revealed the effect of miR‐516a on the dynamic changes characterizing MMP9 degradation. Thus, miR‐516a in T24 cells with low MMP9 expression was overexpressed (Figure 5C) to further investigate the effect of miR‐516a on the degradation rate of MMP9 protein. miR‐516a overexpression could significantly reduce the degradation rate of MMP9 protein in T24 cells compared with control cells (Figure 5D). The inhibition of miR‐516a significantly increased the rate of MMP9 degradation compared to its degradation in the vector cells, indicating that miR‐516a reduced MMP9 degradation (Figures 5E and 5F). MMP9 degradation was decreased in T24T (anti‐miR‐516a/shPHLPP2 #2 & #5) cells than in T24T (anti‐miR‐516a/ sh control) cells, but it returned to the normal rate in T24T (anti‐miR‐516a) cells. Similar findings were obtained in UMUC3 cells (Figures 5G and 5H).

Protein homeostasis is responsible for basic cellular functions, such as the regulation of the level of key enzymes and the removal of abnormal proteins.^30^ The main intracellular protein degradation pathways are the ubiquitin‐proteasome and autophagolysosomal pathways. Because BAF does not affect the role of miR‐516a in BC cell migration and invasion, the possibility that miR‐516a regulates the stability of MMP9 through the autophagolysosomal pathway was excluded. Therefore, T24T/UMUC3 (vector) and T24T/UMUC3 (anti‐miR‐516a) cells were treated with or without the proteasome inhibitor MG‐132 (20 μM) for 10 h to investigate whether the protein degradation of MMP9 depended on the proteasome pathway. Surprisingly, MG‐132‐treated cells exhibited a significant MMP9 protein accumulation compared to its accumulation in the untreated controls (Figures 5I and 5J). Consistent with this, T24T/UMUC3 (anti‐miR‐516a) cells harboring a stable knockdown of PHLPP2 accumulated a significant amount of MMP9 after MG‐132 treatment (Figures 5K and 5L). Overall, these results demonstrated that miR‐516a inhibited the degradation of MMP9 protein in a proteasome‐dependent manner.

### 2.6 SMURF1 is a miR‐516a/PHLPP2 downstream effector that increases MMP9 degradation

**FIGURE 6:**
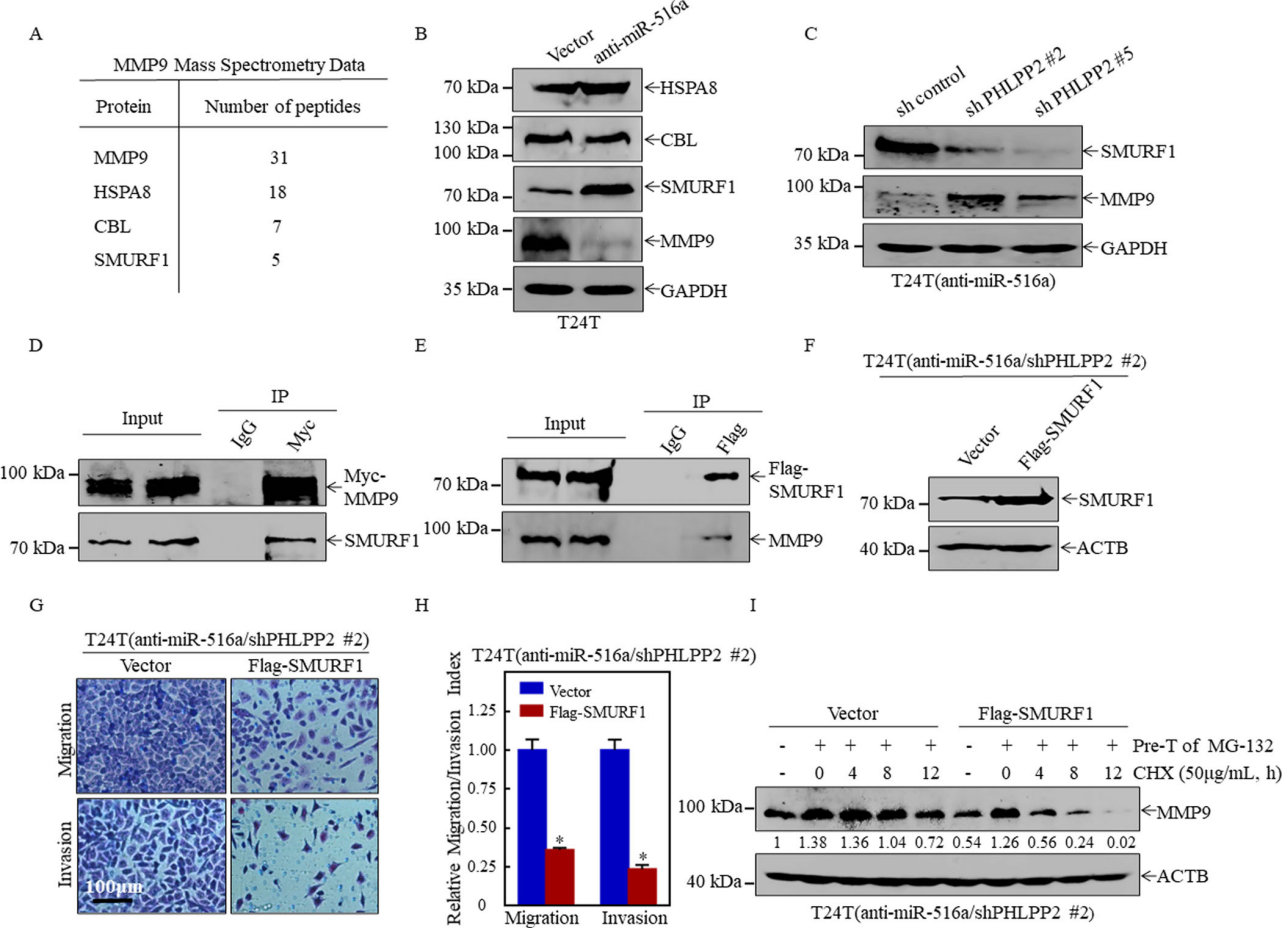
SMURF1 bound MMP9 to promote its degradation, thereby inhibiting BC migration and invasion. (A) Mass spectrometric analysis was performed to identify MMP9‐associated proteins. (B and C) Expression of HSPA8, CBL, SMURF1, and MMP9 in the indicated cell extracts, as determined by western blotting. (D and E) Immunoprecipitation was performed on 293T cell lysates with control IgG (D), anti‐Myc, or (E) anti‐FLAG antibody. The immunoprecipitate was assayed for MMP9 and SMURF1 levels. (F) T24T (anti‐miR‐516a/shPHLPP2 #2/vector) cells and T24T (anti‐miR‐516a/shPHLPP2 #2/Flag‐SMURF1) cells were lysed, and overexpression efficiency of SMURF1 was evaluated by western blotting. (G and H) Invasion and migration of T24T (anti‐miR‐516a/shPHLPP2 #2/vector) and T24T (anti‐miR‐516a/shPHLPP2 #2/Flag‐SMURF1) cells were evaluated by transwell assay. Data were expressed as mean ± SD, and the asterisk (*) indicates a significant difference. (I) The degradation rate of MMP9 was monitored in T24T (anti‐miR‐516a/shPHLPP2 #2/vector) and T24T (anti‐miR‐516a/shPHLPP2 #2/Flag‐SMURF1) cells by western blotting. ImageJ was used to quantify MMP9 expression relative to ACTB

To identify which E3 ubiquitin ligase is involved in regulating the degradation of MMP9 by miR‐516a, we first screened a series of candidates using UbiBrower database^31^ (Figure S3). MMP9 was then subjected to immunoprecipitation and mass spectrometry was performed to determine which E3 ubiquitin ligase plays a role in miR‐516a regulation of MMP9 degradation (Figure 6A). The UbiBrowser predictions and mass spectrometry results suggested that three enzymes, HSPA8, CBL, and SMURF1, could be involved in the regulation of MMP9 degradation by miR‐516a. Hence, the expression of these three proteins was assessed in T24T (vector) and T24T (anti‐miR‐516a) cells. Only the expression of SMURF1 was markedly upregulated in T24T (anti‐miR‐516a) cells, whereas the expression of the other proteins was unchanged (Figure 6B). Consistent with this, SMURF1 expression was much lower in T24T (anti‐miR516a/ shPHLPP2 #2 & #5) cells than in the corresponding vector controls under the same experimental conditions (Figure 6C), suggesting that SMURF1 might participate in the regulation of MMP9 degradation. In addition, the SMURF1‐MMP9 interaction by co‐immunoprecipitation was confirmed. Myc‐MMP9 co‐immunoprecipitation with SMURF1 was found (Figure 6D), and reciprocal immunoprecipitation with Flag‐SMURF1 also brought down MMP9 (Figure 6E), suggesting that MMP9 interacted with SMURF1 in cells.

Next, T24T (anti‐miR516a/shPHLPP2 #2) cells were stably transfected with Flag‐SMURF1 or a control plasmid (Figure 6F) to confirm the role of SMURF1 in miR‐516a‐mediated promotion of BC migration and invasion. Ectopic expression of Flag‐SMURF1 restored the ability of migration and cell invasion in BC cells (Figure 6G and 6H). Hence, the effect of SMURF1 overexpression was examined on the rate of MMP9 degradation. As shown in Figure 6I, MMP9 was accumulated in a similar amount in T24T (anti‐miR516a/shPHLPP2 #2/vector) and T24T (anti‐miR516a/shPHLPP2 # 2/Flag‐SMURF1) cells pretreated with the proteasome inhibitor MG‐132. After the removal of MG‐132, the effect of SMURF1 on the rate of MMP9 degradation was monitored after the addition of CHX at specified time points. MMP9 degradation was dramatically increased by SMURF1 overexpression compared to vector control cells. Together, our results strongly indicated that MMP9 degradation and the miR‐516a‐specific promotion of BC metastasis were regulated by the miR‐516a downstream effector SMURF1.

### 2.7 AKT/FOXO3A signaling pathway mediates the regulation of SMURF1 transcription by miR‐516a/PHLPP2

**FIGURE 7:**
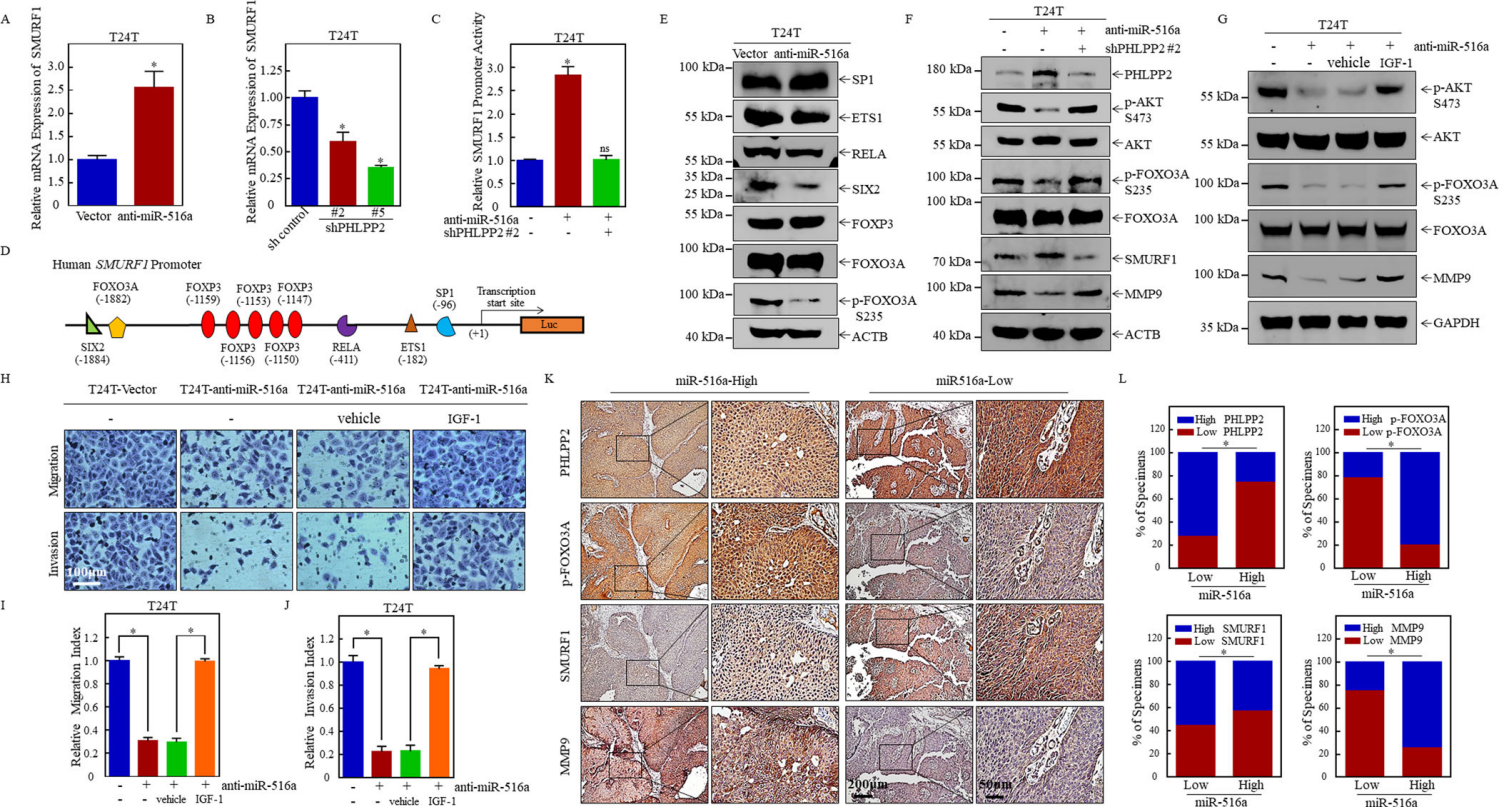
MiR‐516a/PHLPP2 promoted BC migration and invasion via AKT/FOXO3A/SMURF1 signaling pathway. (A and B) *SMURF1* mRNA expression, as determined by real‐time PCR. (C) Luciferase activity was evaluated after *SMURF1* promoter plasmid and pRL‐TK were transiently co‐transfected into the indicated cells. (D) Bioinformatics software was used to predict transcription factor binding sites of human *SMURF1* promoter. (E‐G) The indicated proteins were analyzed by western blotting. (H‐J) Transwell assay was performed in T24T (Vector), T24T (anti‐miR‐516a), T24T (anti‐miR‐516a, vehicle) and T24T (anti‐miR‐516a, IGF‐1) cells. (K) Immunohistochemical staining of PHLPP2, p‐FOXO3A, SMURF1, and MMP9 in 71 BC clinical specimens; 100× magnification, scale bar: 200 μm; 400× magnification, scale bar: 50 μm. (L) Correlation between miR‐516a and PHLPP2/p‐FOXO3A/SMURF1/MMP9 in 71 clinical BC specimens

SMURF1 mRNA expression was first monitored to elucidate the mechanism used by miR‐516a to downregulate SMURF1 expression. The *SMURF1* mRNA level was significantly higher in T24T (anti‐miR‐516a) cells than in T24T (vector) cells (Figure 7A). Consistent with this, inhibition of PHLPP2 in T24T (anti‐miR‐516a) cells decreased the *SMURF1* mRNA level (Figure 7B). These results suggested that the regulation of SMURF1 expression by miR‐516a might occur at the transcriptional level. Moreover, the luciferase reporter assay showed that miR‐516a inhibition caused an approximately 2.8‐fold increase in SMURF1‐driven promoter activity, and the knockdown of PHLPP2 in T24T (anti‐miR‐516a) cells restored *SMURF1* promoter activity (Figure 7C).

Potential transcription factors binding the SMURF1 promoter region were then investigated. Our bioinformatics analysis revealed several potential transcription factors, including FOXO3A, FOXP3, RELA, ETS‐1, and SP1 (Figure 7D). In addition, a ChIP assay previously reported revealed that SIX2 can directly bind the CAGCTG sequence in the *SM*URF1 promoter,^32^ and another study suggested that NF‐κB specifically binds to the –411 to –420 region of the SMURF1 promoter and regulates its activity. Next, western blot was performed to detect the expression of these transcription factors in T24T (vector) and T24T (anti‐miR‐516a) cells. Only p‐FOXO3A S253 and SIX2 were remarkably downregulated, whereas the levels of FOXO3A, FOXP3, NF‐κB (RELA), ETS‐1, and SP1 were unchanged, indicating that p‐FOXO3A S253 and SIX2 might be involved in miR‐516a‐mediated regulation of *SMURF1* transcription (Figure 7E).

Because SIX2 can upregulate SMURF1 expression at gene transcription level, which was the opposite of our result, SIX2 was excluded as a candidate transcription factor in miR‐516a‐mediated regulation of *SMURF1* expression. Although the total FOXO3A protein level did not change after miR‐516a inhibition, p‐FOXO3A Ser253 protein expression was significantly reduced. This result implies that the expression of nonphosphorylated FOXO3A was upregulated, in turn suggesting that FOXO3A regulated the transcription of *SMURF1*. Protein kinase B (also known as AKT) is an important enzyme that directly phosphorylates FOXO3A. Specifically, Ser253 phosphorylation of AKT regulates the nuclear/cytoplasmic shuttle of FOXO3A. The activation of AKT promotes the binding of FOXO3A to nuclear export proteins, hiding the nuclear localization signals and preventing FOXO3a from re‐entering the nucleus.^33^, ^34^, ^35^ Hence, we hypothesized that AKT/FOXO3A signaling might be activated by the upregulation of miR‐516a. As shown in Figure 7F, the phosphorylation of both FOXO3A and AKT increased in T24T (vector) and T24T (anti‐miR‐516a/shPHLPP2#2) compared to their phosphorylation in T24T (anti‐miR‐516a/sh control) cells. Such results validate our hypothesis that miR‐516a‐PHLLP2 regulated the transcription of SMURF1 through the AKT‐FOXO3A signaling pathway, thereby regulating the stability of MMP9 protein. In order to further clarify the role of AKT‐FOXO3A pathway in miR‐516a‐meidated promotion of the migration and invasion of BC cells, we used AKT activator insulin‐like growth factor‐1 (IGF‐1) to treat T24T anti‐miR‐516a cells, and found that IGF‐1 treatment can significantly reverse the inhibitory effect of miR‐516a on the AKT‐FOXO3A pathway as well as the migration and invasion of BC (Figure 7G‐J). The expression of miR‐516a, PHLPP2, p‐FOXO3A, SMURF1, and MMP9 in clinical specimens were further detected to elucidate their clinical relevance. The results showed that the expression of PHLPP2 and SMURF1 were significantly and negatively correlated with the expression of miR‐516a in 71 pairs of clinical specimens, whereas the expression of p‐FOXO3A and MMP9 were significantly and positively correlated with the expression of miR‐516a (Figures **7**K and **7**L). Together, miR‐516a does promote BC cell migration and invasion by activating the AKT‐FOXO3A pathway.

## 3 DISCUSSION

The majority of miRNA genes are located in cancer‐related genomic regions or vulnerable sites, and dysregulation in their expression promotes cancer development.^36^ MiR‐516a belongs to the C19MC and has been described as an oncogene in some studies. It promotes the formation of abdominal aortic aneurysms,^37^ and risk stratification in patients with neuroblastoma improves when miR‐487b and miR‐516a‐5p are both expressed, suggesting that miR‐516a‐5p plays a carcinogenic role.^7^ However, several other reports showed that miRNAs from C19MC also have tumor‐suppressive functions in some cancers. For example, miR‐516a‐5p targets HIST3H2A to inhibit nonsmall cell lung cancer proliferation.^38^ This situation seems contradictory, and is not unique to C19MC miRNAs. The behavior of these miRNAs is under the control of the cellular environment, resulting in dual functions as oncogenes and tumor suppressors; thus, these miRNAs are classified as “context‐dependent.”^10^ The results of this study suggested that the overexpression of miR‐516a promoted the proliferation and metastasis of BC cells. Thus, miR‐516a played oncogenic roles in BC, indicating that this miRNA might be a potential diagnostic and prognostic biomarker and a therapeutic tool.

Our research previously published revealed that miR‐516a promotes the proliferation of BC cells in vivo and in vitro by inhibiting the autophagy of BC cells.^13^ Other studies showed that autophagy is also involved in the metastasis of malignant tumors in many aspects. Unexpectedly, our experimental results in the present work showed that miR‐516a did not regulate the migration and invasion of BC cells through autophagy, and our further experiment revealed that miR‐516a regulated the migration and invasion of BC cells through autophagy‐independent pathways. This result also indicated that miR‐516a and its target gene PHLPP2 might be relatively independent in regulating the proliferation and metastasis of BC cells.

MMP9, a member of the zinc‐dependent proteolytic enzymes, is essential for local proteolysis of the extracellular matrix and tumor metastasis.^39^ Studies have shown that MMP9 can not only degrade the extracellular matrix and basement membrane, and promote the invasion of cancer cells, but also play a key role in the process of epithelial‐mesenchymal transition to promote cell motility. It can be seen that MMP9 can promote tumor cell invasion and metastasis through various comprehensive effects.^40^ Many signaling pathways take part in the regulation of MMP9 expression, but the regulation takes place primarily at the transcriptional level.^41^, ^42^, ^43^ However, few studies described the regulation of MMP9 degradation.^44^ The UbiBrowser database prediction and mass spectrometry identification revealed that SMURF1, an E3 ubiquitin protein ligase, regulates MMP9 degradation. Further experiments are needed to confirm the details of SMURF1 binding to MMP9 and promoting its degradation.

SMURF1 is an E3 ubiquitin ligase that regulates cell differentiation, cell shape and polarity, cell adhesion and migration, autophagy, embryonic pattern formation, morphogenesis, organogenesis, and biological physiology and fertility. According to recent studies, SMURF1 plays dual roles in tumors, acting as both a tumor promoter and suppressor.^45^, ^46^ SMURF1‐mediated RhoA degradation is vital for cancer cell metastasis.^47^ On the other hand, excessive degradation of RhoA also plays a role in inhibiting tumor invasion.^48^ Besides RhoA, SMURF1 can also target hPEM‐2^49^ and talin^50^ for ubiquitin‐mediated proteasome degradation during metastasis. This study demonstrated that SMURF1 bound MMP9 to promote its degradation, thereby limiting the migration and invasion of BC cells. In our subsequent research, the specific structure and type of MMP9 ubiquitination induced by SMURF1 will be explored.

In this study, the results revealed the specific mechanism involved in miR‐516a‐mediated promotion of BC cell migration and invasion, also involved in the downregulation of the miR‐516a target gene *PHLPP2*. The further downregulation of SMURF1 expression attenuated MMP9 degradation, and ultimately promoted BC cell migration and invasion. Our investigation to explore how PHLPP2 regulates the expression of SMURF1 revealed that p‐FOXO3A S253 affected the transcription of *SMURF1* in stably transfected cell lines in which miR‐516a was suppressed. FOXO3A, also known as FKHRL‐1, is a member of the Forkhead transcription factor family. AKT phosphorylates FOXO3A, resulting in impairment of cytoplasmic retention and FOXO3a nuclear transcriptional activity.^51^, ^52^ PHLPP2 inhibits AKT activity by dephosphorylation of Ser‐473, a key regulatory site in AKT.^53^ Surprisingly, our results showed that miR‐516a indeed activated the AKT/FOXO3A pathway by targeting PHLPP2, thereby promoting BC cell metastasis. Many studies linked the functions of PHLPP2, AKT, and FOXO3A to cell survival and apoptosis, but few reports linked these factors to metastasis.^21^, ^34^, ^54^ Our findings revealed that the migration and invasion of BC cells are regulated via the PHLLP2/AKT/FOXO3A signaling axis, representing a novel target in the treatment of BC.

In conclusion, our work demonstrated that miR‐516a promoted BC metastasis by regulating PHLPP2/p‐AKT/p‐FOXO3A/SMURF1/MMP9, and that PHLPP2 played a key role in this pathway. These findings might provide a deeper understanding of the molecular mechanisms of miR‐516a‐induced BC metastasis, and new potential targets in the prevention and treatment of BC.

## 4 MATERIALS AND METHODS

### 4.1 Reagents, antibodies, and plasmids

BAF was purchased from Selleck Chemicals (S1413; Houston, TX, USA). CHX was purchased from Calbiochem (San Diego, CA, USA). IGF‐1 was purchased from Hangzhou Multi Sciences (PK002‐01, Hangzhou, Zhejiang, China). The shRNA plasmid specifically targeting PHLPP2 was purchased from Open Biosystems (Thermo Fisher Scientific, NY, USA). Hsa‐miR‐516a‐5p inhibitor (anti‐miR‐516a) and overexpression plasmids were purchased from Shanghai GenePharma (C6133). Human *SMURF1* cDNA was cloned into pcDNA3.1 (P0157; Miaolingbio.com, Wuhan, Hubei, China) using the following primers with *Eco*RI and *Xho*I linkers: (F) 5′‐TAG CGA ATT CGC CAC CAT GTC GAA CCC CGG GAC ACG CAG GAA C‐3′, (R) 5′‐TAG CCT CGA GCG ATC CGC CTC CAC CCT CCA CAG CAA ACC CGC AGG TCT CCT C‐3′. Human *MMP9* cDNA was cloned into pcDNA3.1 using the following primers with *Kpn*I and *Eco*RI linkers: (F) 5′‐TAG CGG TAC CGC CAC CAT GAG CCT CTG GCA GCC CCT GGT CCT GGT G‐3′, (R1) 5′‐AGC TTC TGC TCA TGG TGA TGG TGG TGA TGG TCC TCA GGG CAC TGC AGG ATG TCA TAG GTC‐3′, and (R2) 5′‐ TAG CGA ATT CAC AGG TCC TCC TCT GAG ATC AGC TTC TGC TCA TGG TGA TGG TGG TGA TGG‐3′. The primers (F) 5′‐ATT GGT ACC TAC TAA CGG GAT TGG CAG AAG‐3′ and (R) 5′‐ AAT AAG CTT TGC CGC CTC TCC GAG CCG CGC G‐3′ were used to construct a human *SMURF1* gene promoter (–2000) in the pGL3‐basic vector (E1751; Promega Corporation, Madison, WI, USA). Antibodies against RhoA (2117), RhoC (3430), Rac123 (2465), CDC42 (2466), Myc (2276), SMURF1 (2174), FALG (8146), AKT (4685), p‐AKT S473 (4046), FOXO3A (12829), p‐FOXO3A S253 (9466), and SP1 (9389) were purchased from Cell Signaling Technology (Boston, MA, USA). Antibodies against MMP2 (sc‐10736), MMP9 (sc‐393859), SIX2 (sc‐377193), HSPA8 (sc‐7298), CBL (sc‐1651), and ETS‐1 (sc‐350) were purchased from Santa Cruz Biotechnology (Dallas, TX, USA). The antibody against RELA (ab16502) and PHLPP2 (ab77665) was purchased from Abcam (Cambridge, UK). The FOXP3 (22731‐1‐AP) antibody was purchased from Proteintech Group, Inc (Rosemont, IL, USA). Antibodies against GAPDH (Ab0037) and ACTB (Ab0011) were purchased from Abways Technology (Shanghai, China).

### 4.2 Cell culture and BC tissue collection

DMEM (11995‐065, Gibco/Thermo Fisher Scientific) was used to culture UMUC3 cells (CRL‐1749; ATCC, Rockefeller, MD, USA). Dr Dan Theodorescu (University of Colorado Comprehensive Cancer Center, Denver, CO, USA) generously provided us the T24T and T24 cell lines, which were maintained in Dulbecco's modified Eagle's medium (DMEM):F12 (1:1) (10565‐018; Gibco/Thermo Fisher Scientific) supplemented with 5% fetal bovine serum (FBS; 1750114, Gibco/Thermo Fisher Scientific) and incubated in a humidified atmosphere of 5% CO_2_ at 37°C. Human BC samples were obtained from the bladders of 71 patients who underwent transurethral resection of bladder tumor or radical cystectomy at the Wenzhou Medical University Affiliated First Hospital or Second Affiliated Hospital of Wenzhou Medical University between 2016 and 2019. Patient name, gender, age, case number, and pathological information were collected. The authorized case information is shown in Table S1. Preoperative biopsy and postoperative pathological examination of the above patients confirmed bladder urothelial carcinoma; liquid nitrogen irrigation was used to freeze the specimens as soon as they were collected. All experiments related to clinical specimens obtained the approval of the Ethics Committee of Wenzhou Medical University.

### 4.3 RNA isolation and real‐time PCR

Total RNA was isolated from tissue samples or cultured cells using Qiazol reagent (Qiagen, Gaithersburg, MD, USA) or TRIzol reagent (Invitrogen, Carlsbad, CA, USA). RNA was reverse‐transcribed into miRNA cDNA or total cDNA using the Prime Script miRNA cDNA synthesis kit (Qiagen) or PrimeScript RT kit (Qiagen), and then subjected to quantitative RT‐PCR (qRT‐PCR). mRNA or miRNA expression was determined by qPCR on a Q6 real‐time PCR System (Applied Biosystems, Carlsbad, CA, USA) using SYBR Green Master Mix (4309155, Applied Biosystems) or the miScript PCR Kit (Qiagen), using *GAPDH* or *U6*, respectively, as the internal loading control. Reverse transcription and PCR were performed as described in our previous report.^55^ Specific primers for human *MMP9* (Forward: 5′‐TTC CAA ACC TTT GAG GGC GA‐3′ and Reverse: 5′‐CTG TAC ACG CGA GTG AAG GT‐3′), human *SMURF1* (Forward: 5′‐AAC TGA AAC CCA ATG GCA GAA ATGT‐3′ and Reverse: 5′‐TTG CCA GAA CCA CCG CAC GAT G‐3′), and human *GAPDH* (Forward: 5′‐GAC TCA TGA CCA CAG TCC ATG C‐3′, Reverse: 5′‐CAG GTC AGG TCC ACC ACC ACT GA‐3′) were used for PCR amplification. The primer for miR‐516a (5′‐TGC TTC CTT TCA GAG GGT‐3′) was synthesized by Sunny Biotechnology (Shanghai, China), and *U6* was used as the internal loading control through the primer provided in the miScript PCR Kit. The data were analyzed as previously described.^55^

### 4.4 Western blot analysis

Cells were lysed in boiling buffer^56^ and then sonicated; the protein concentration was measured on a NanoDrop One (Thermo Fisher Scientific), and then the volume was adjusted to bring the concentration to a consistent level across all samples. Total proteins were separated by SDS‐PAGE, and then the protein imprints were transferred to a PVDF membrane and blocked in 5% skim milk at room temperature for 1 h. The membrane was incubated overnight with the primary antibody at 4°C and then incubated with the secondary antibody for 3 h. ECF developing reagent (RPN5785, GE Healthcare, Boston, MA, USA) was diluted at the appropriate ratio with TBS, and the membrane was scanned on a Typhoon fluorescence imager (GE Healthcare).

### 4.5 Cell migration and invasion assay

After the migration device (353097, Corning Incorporated, Corning, NY, USA) and invasion device (354480, BD Biosciences, Bedford, MA, USA) were subjected to room temperature equilibration for 10 min, the two devices were incubated for 2 h in a 37°C incubator, and 400‐μL serum‐free DMEM was added in the upper chamber and 700 μL in the lower chamber. Cells (6 × 10^4^ in 400 μL) in 0.1% FBS medium with or without 1 nM BAF were seeded in a Transwell apparatus, and the lower chamber was filled with complete cell culture medium (700 μL) with or without BAF (1 nM). After incubation for 24 h, the cells were fixed with 4% PFA for 15 min, transferred to 100% methanol for 20 min, and finally stained with Giemsa (diluted 1:10 with PBS) for 30 min at room temperature in the dark. Images were acquired on a DP71 microscope (Olympus America, Center Valley, PA, USA). Invasion and migration were quantified by counting five fields per chamber. Data are shown as mean ± standard deviation of three independent repeated experiments.

### 4.6 Lung metastatic assay

All animal experiments were approved by the Animal Ethics Committee of the Animal Laboratory of Wenzhou Medical University, and the lung metastasis test in nude mice was performed in the SPF environment of the Animal Center of Wenzhou Medical University. Female athymic mice, 3‐4 weeks’ old, were purchased from GemPharmatech (license number: SCXK [SU] 2018‐0008; Nanjing, Jiangsu, China). After acclimation for 1 week, the nude mice were ear‐tagged and randomly divided in two cages. After the growing of T24T (vector) and T24T (anti‐miR516a) cells, 3 × 10^6^ cells in 100‐μL PBS were injected into each mouse via the tail vein. The mice were then raised normally for 6‐8 weeks. The nude mice were sacrificed by euthanasia, and their lungs were removed and fixed in Bouin's solution (Sigma Aldrich, St. Louis, MO, USA) for 24‐48 h. Next, the images were acquired to count the number of metastases, and then, the lung tissue was embedded in paraffin, and cut in slices that were subjected to hematoxylin‐eosin staining.

### 4.7 Liver metastasis animal model

After the nude mice were anesthetized, the peritoneum was open and the spleen was gently pulled out. Then 5 × 10^6^ T24T (transfected with anti‐miR‐516a) or vector control cells in 200‐μL PBS were slowly injected into the spleen, this process lasts for 3‐5 min, and the abdominal wall was sutured with full layer. Mice were sacrificed at a specified time (6 weeks) after cell injection to determine the number and size of liver metastases.

### 4.8 Luciferase reporter assay

T24T (vector), T24T (anti‐miR‐516a/sh control), and T24T (anti‐miR‐516a/shPHLPP2 #2) cells were transiently co‐transfected with *SMURF1* promoter plasmid and pRL‐TK (E2241, Promega). *SMURF1* promoter activity was measured after 24 h by the Dual‐Glo Luciferase Assay Kit (E1960, Promega) using TK as the internal reference.

### 4.9 Immunoprecipitation

After transient transfection for 24 h, 293T cells were washed with PBS and lysed on ice for 15 min in 1‐mL cell lysis buffer (1:10 dilution, 9803; Cell Signaling Technology) containing complete protein cocktail inhibitor (04693116001; Roche, Basel, Switzerland). Centrifugation was performed to clarify the cell lysates. The lysates were incubated at 4°C overnight with the indicated antibody and then incubated for additional 2 h with Protein A/G agarose (sc‐2003, Santa Cruz Biotechnology). After extensive washing with lysis buffer, the immunocomplexes were analyzed by immunoblotting or detected by mass spectrometry, which was performed by Guangzhou FitGene Biotechnology.

### 4.10 Immunohistochemistry

The clinical paraffin‐embedded sample was cut into 5‐μm‐thick sections, and 100% xylene was used to deparaffinize the sections. The tissue was hydrated with gradient alcohols and then water, and the antigen retrieval solution was performed in a microwave oven for 30 min. Next, the slides were treated with 3% H_2_O_2_ to block endogenous peroxidase, and incubated overnight with the primary antibodies PHLPP2 (sc‐393859, Santa Cruz biotechnology), p‐FOXO3A (ST49‐01, ThermoFisher), SMURF1 (55175‐1‐AP, Proteintech), and MMP9 (sc‐393859, Santa Cruz biotechnology) at 4°C. After washing, the sections were incubated with poly‐HRP‐conjugated anti‐Rabbit IgG (SA1022, BOSTER) or poly‐HRP‐conjugated anti‐Mouse IgG (SA1021, BOSTER) for 60 min at 37°C. The sections were visualized with DAB (56990, Abcam), hematoxylin was used to counterstain, and the gradient dehydration with alcohols and xylene was performed. Finally, the sections were mounted using neutral gum and observed under a microscope.

### 4.11 Statistical analysis

Statistical analysis was performed using the GraphPad Prism 5.0 statistical software. Experimental data were expressed as mean ± standard deviation. The Student's *t*‐test was used to evaluate differences between two groups, and the log‐rank test was used to analyze the differences in survival. A value of *p* ≤ 0.05 was considered statistically significant relative to the control.

## AUTHOR CONTRIBUTIONS

Haishan Huang, Xing Huang, and Honglei Jin conceived and designed the study. Yuanyuan Chang, Jiugao Ma, Zhijian Zheng, Binuo Sun, Yiting Lyu, Mengqi Lin, He Zhao, Yongyong Lu, Hongyan Li, and Honglei Jin evaluated the biological function of the cells, performed the RT‐PCR assay and transwell assay, and conducted the statistical analysis. Yuanyuan Chang, Honglei Jin, Zhijian Zheng, Binuo Sun, Yiting Lyu, Mengqi Lin, and He Zhao performed the animal experiments. Gang Zhang and Yuanyuan Chang performed the bioinformatic analysis. Yongyong Lu and Qipeng Xie collected the clinical samples. Hongyan Li performed immunohistochemistry assay and the statistical analysis. Yuanyuan Chang, Xing Huang, and Haishan Huang drafted the manuscript. Yuanyuan Chang, Hongyan Li, Xing Huang, and Haishan Huang revised the manuscript. All authors read and approved the final version of the manuscript.

## ETHICS APPROVAL AND CONSENT TO PARTICIPATE

All experiments related to clinical specimens were approved by the ethics committee of Wenzhou Medical University. All animal experiments were performed in accordance with the regulations of the Experimental Animal Ethics Committee of Wenzhou Medical University.

## CONFLICT OF INTEREST

The authors declare no conflict of interest.

## AVAILABILITY OF DATA AND MATERIALS

The datasets used in this study are available from corresponding authors on a reasonable request.

## Supporting information


**Supplemental Table 1**. BC patients’ information including case number, medical record number, gender, age, and tumor stage.

**Supplemental Table 2**. MiR‐516a promoted T24T cell lung metastasis in nude mice.

**Supplemental Table 3**. MiR‐516a promoted T24T cell liver metastasis in nude mice.

**Supplemental Figure 1**. MiR‐516a expression of BC tissues increased inpatients with metastasis in comparison to non‐metastatic patients according to the TCGA database.

**Supplemental Figure 2**. The binding of miR‐516a to the 3′‐UTR of PHLPP2 in T24 and T24T cells.

**Supplemental Figure 3**. E3 ligases potentially involved in MMP9 degradation, predicted by UbiBrowser.
