# Supplementary figures and images for: MiRNA‐516a promotes bladder cancer metastasis by inhibiting MMP9 protein degradation via the AKT/FOXO3A/SMURF1 axis
Source: Clin Transl Med. 2020 Dec 21;10(8):e263. doi: 10.1002/ctm2.263 (PMC7752166; doi:10.1002/ctm2.263)

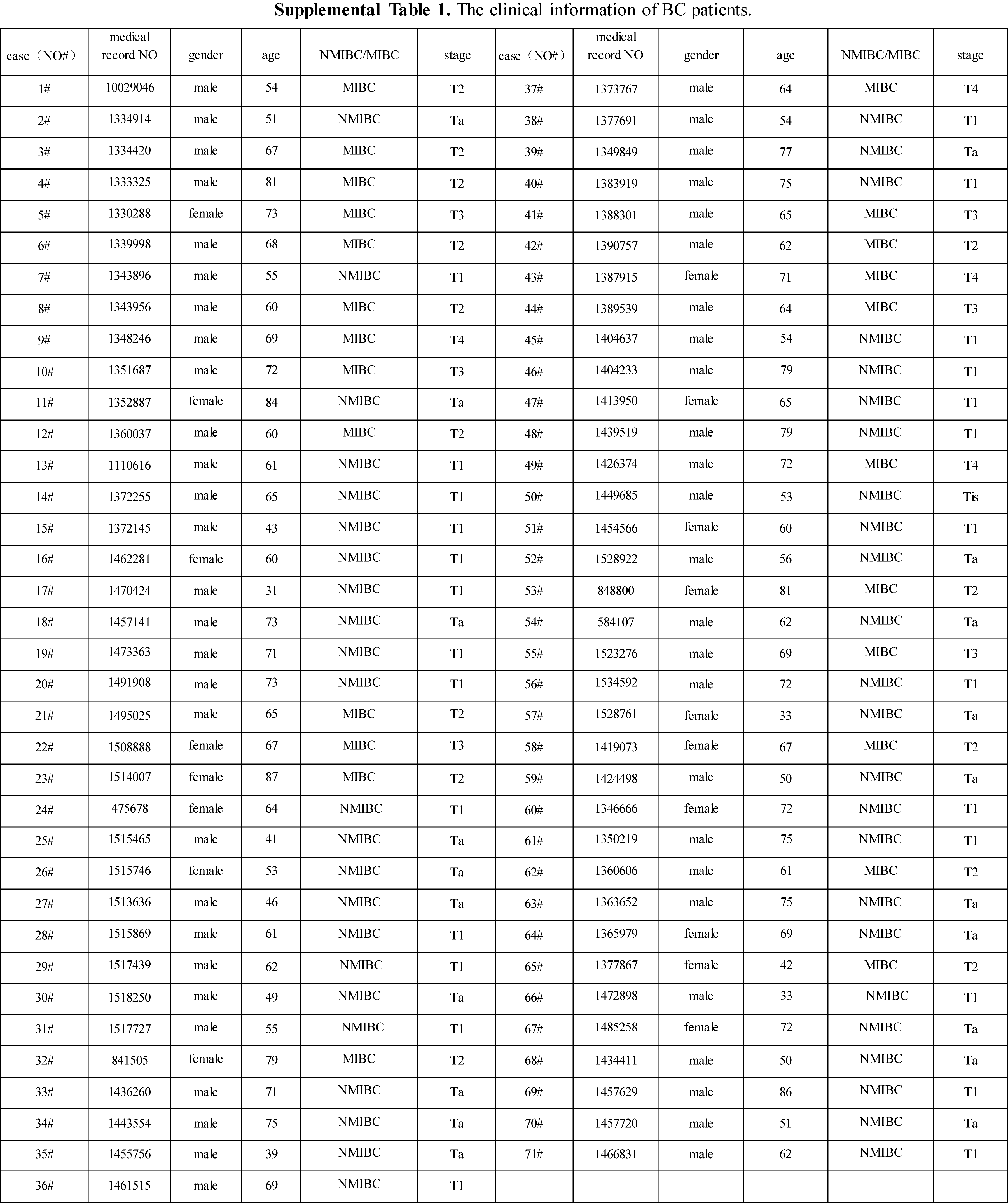

Supplement: Supplementary file 1 — Supplemental Table 1. BC patients’ information including case number, medical record number, gender, age, and tumor stage. [file CTM2-10-e263-s004.tif]

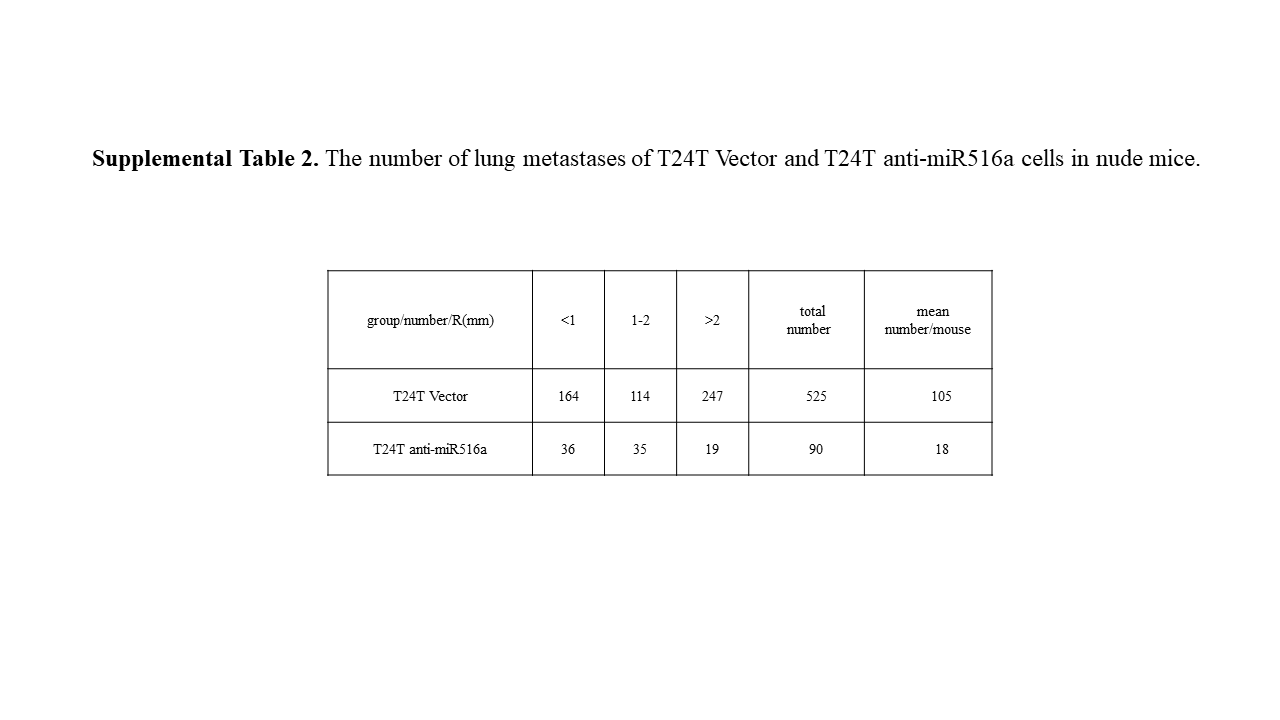

Supplement: Supplementary file 2 — Supplemental Table 2. MiR‐516a promoted T24T cell lung metastasis in nude mice. [file CTM2-10-e263-s005.tif]

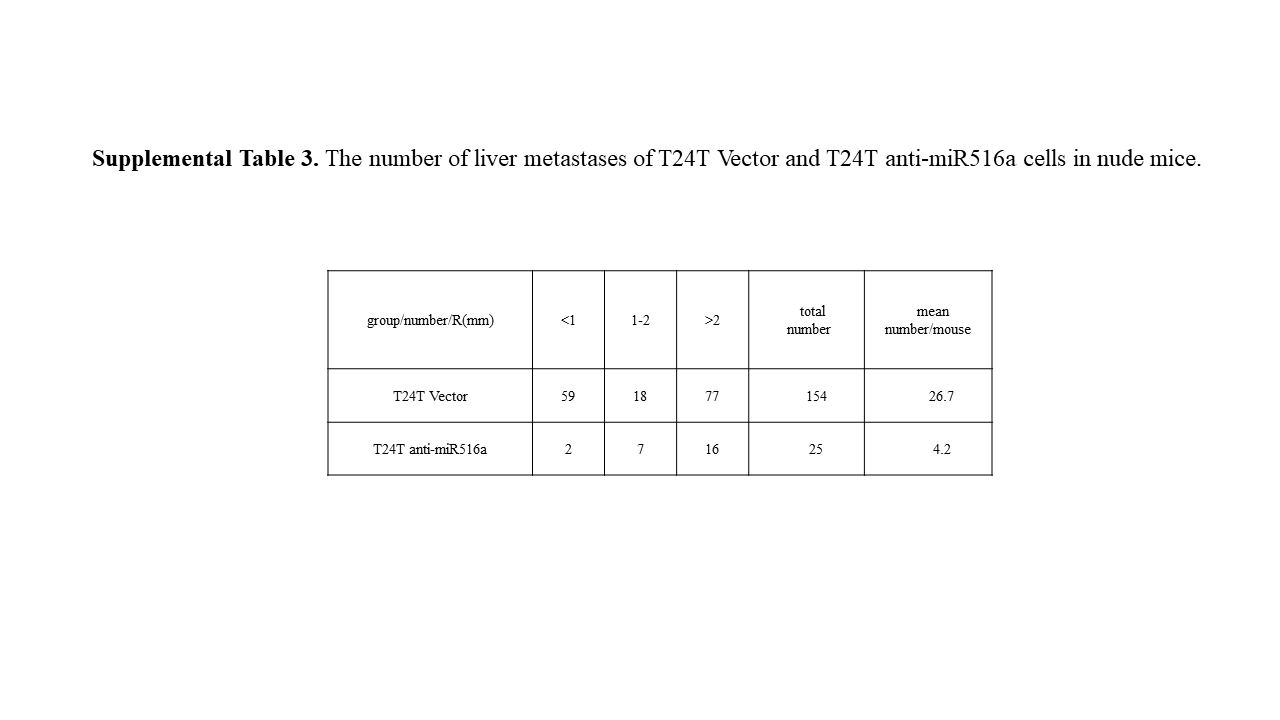

Supplement: Supplementary file 3 — Supplemental Table 3. MiR‐516a promoted T24T cell liver metastasis in nude mice. [file CTM2-10-e263-s006.tif]

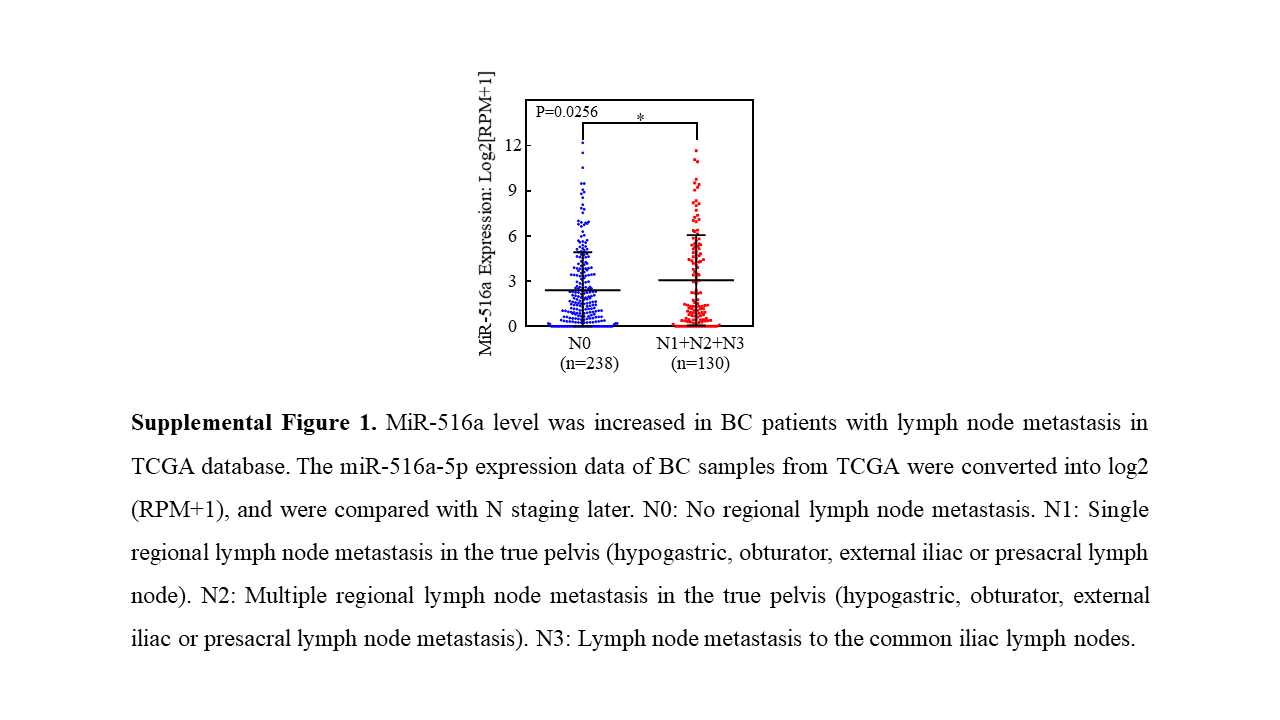

Supplement: Supplementary file 4 — Supplemental Figure 1. MiR‐516a expression of BC tissues increased inpatients with metastasis in comparison to non‐metastatic patients according to the TCGA database. [file CTM2-10-e263-s001.tif]

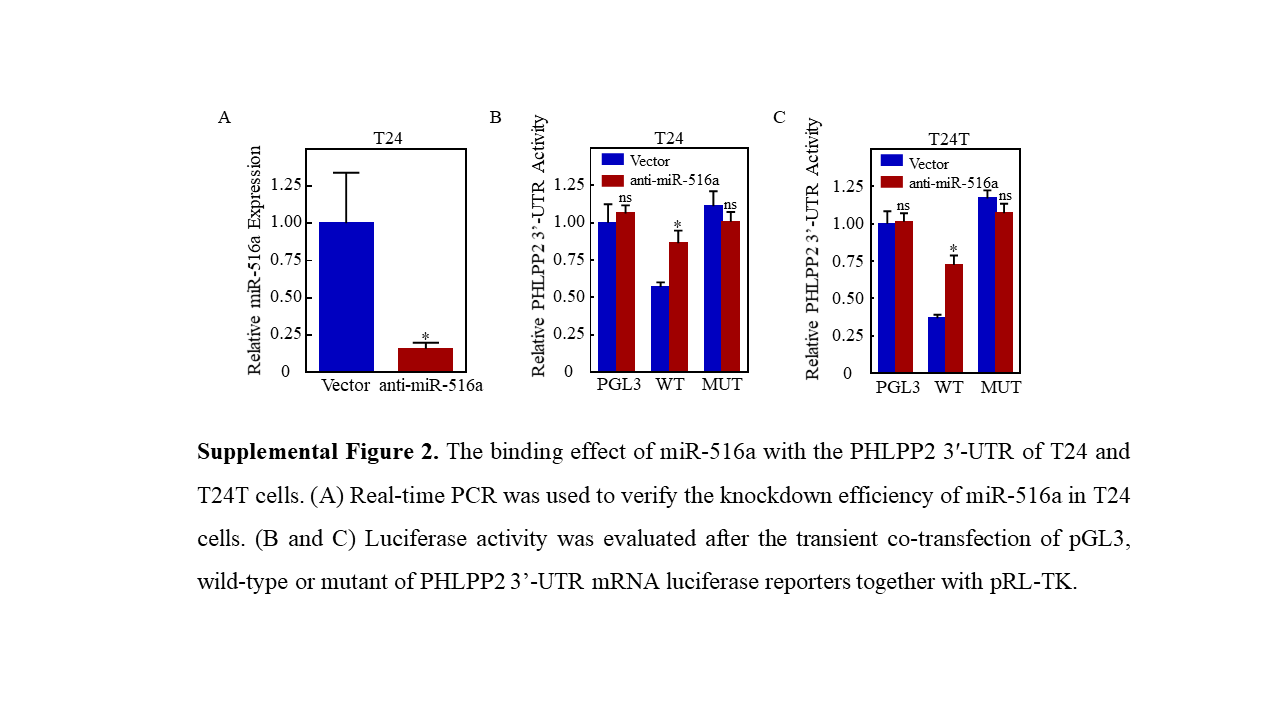

Supplement: Supplementary file 5 — Supplemental Figure 2. The binding of miR‐516a to the 3′‐UTR of PHLPP2 in T24 and T24T cells. [file CTM2-10-e263-s002.tif]

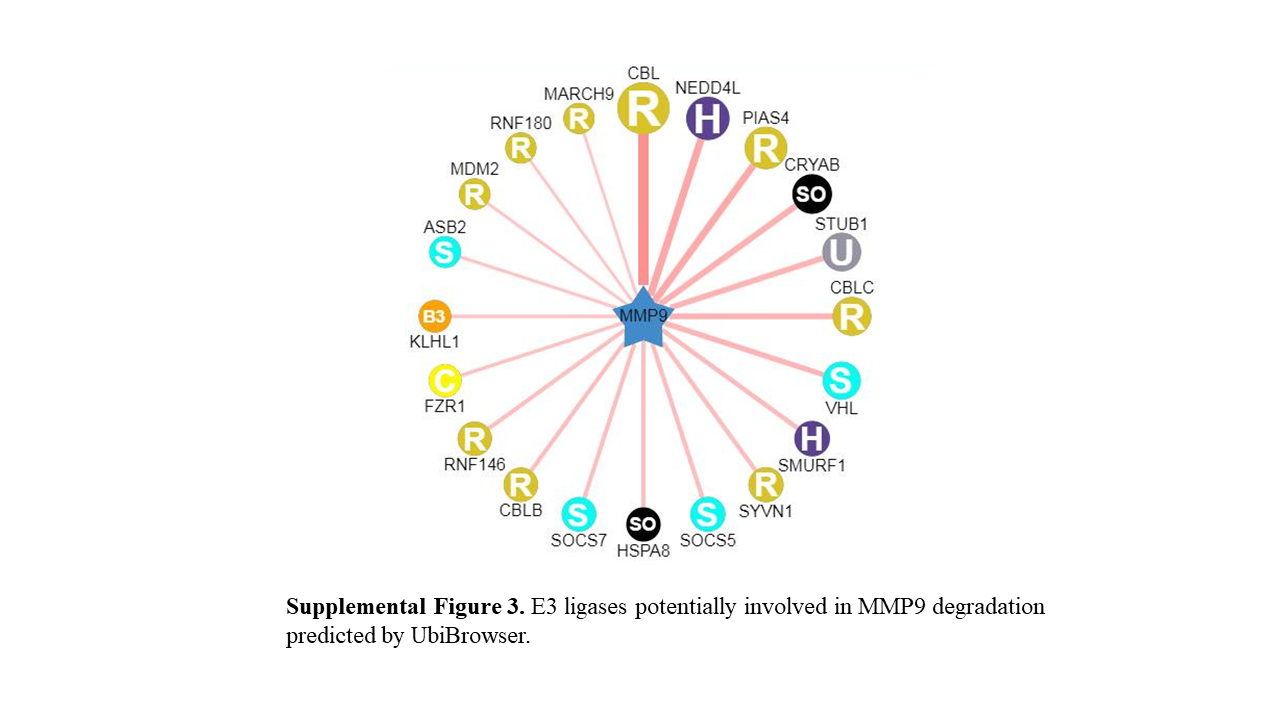

Supplement: Supplementary file 6 — Supplemental Figure 3. E3 ligases potentially involved in MMP9 degradation, predicted by UbiBrowser. [file CTM2-10-e263-s003.tif]
